# Blood pressure variability in individuals with diabetes *mellitus*: a scoping review

**DOI:** 10.1590/0034-7167-2021-0804

**Published:** 2022-05-11

**Authors:** Antonia Fabiana Rodrigues da Silva, Rebeca Chaves Cruz, Nila Larisse Silva de Albuquerque, Viviane Martins da Silva, Thelma Leite de Araujo

**Affiliations:** I Universidade Federal do Ceará. Fortaleza, Ceará, Brazil.; II Universidade da Integração Internacional da Lusofonia Afro-Brazileira. Redenção, Ceará, Brazil.

**Keywords:** Blood Pressure Determination, Blood Pressure, Diabetes *Mellitus*, Adult, Older Adult, Determinación de la Presión Sanguínea, Presión Arterial, Diabetes *Mellitus*, Adulto, Anciano

## Abstract

**Objectives:**

to map methods and devices used to assess very short-, short-, medium-, and long-term pressure variability in adults with type 1 or 2 diabetes *mellitus*.

**Methods:**

scoping review conducted in January and February 2021 in MEDLINE, Web of Science, CINAHL, LILACS, PubMed, and Embase databases. Studies conducted within the last ten years analyzing pressure variability in adult and older patients with diabetes *mellitus* type 1 or 2 were included. Studies that used discontinued devices were excluded.

**Results:**

the sample was composed of 25 articles published since 2017, with the majority developed in Japan (n=11); with the predominance of the oscillometric method (n=22); the most used devices were from the Omron^®^ brand (n=14); the most detected type was long-term variability (n=10).

**Conclusions:**

we observed the increasing application of the oscillometric method for pressure variability analysis with various brands and models of automatic devices.

## RESUMO

**Objetivos:**

mapear métodos e dispositivos utilizados para avaliação da variabilidade pressórica de curtíssimo, curto, médio e longo prazo em adultos com diabetes *mellitus* tipo 1 ou 2.

**Métodos:**

revisão de escopo realizada nos meses de janeiro e fevereiro de 2021 nas bases de dados MEDLINE, *Web of Science*, CINAHL, LILACS, PubMed e Embase. Incluíram-se estudos, realizados nos últimos dez anos, que analisaram a variabilidade pressórica em pacientes adultos e idosos com diabetes *mellitus* tipo 1 ou 2. Excluíram-se estudos que utilizaram dispositivos descontinuados.

**Resultados:**

a amostra foi composta por 25 artigos, publicados a partir de 2017, com maioria desenvolvida no Japão (n=11); predominância do método oscilométrico (n=22); os dispositivos mais utilizados foram da marca Omron^®^ (n=14); o tipo de variabilidade mais detectada foi a de longo prazo (n=10). **Conclusões:** observou-se o crescente uso do método oscilométrico para análise da variabilidade pressórica, apresentando variedade de marcas e modelos de dispositivos automáticos.

## RESUMEN

**Objetivos:**

mapear métodos y dispositivos utilizados para evaluación de la variabilidad presórica de cortísimo, corto, mediano y largo plazo en adultos con diabetes *mellitus* tipo 1 o 2.

**Métodos:**

revisión del área realizada en enero y febrero de 2021 en las bases de datos MEDLINE, Web of Science, CINAHL, LILACS, PubMed y Embase. Incluidos estudios, realizados en los últimos diez años, que analizaron la variabilidad presórica en pacientes adultos y ancianos con diabetes *mellitus* tipo 1 o 2. Excluidos estudios que utilizaron dispositivos descontinuados.

**Resultados:**

la muestra fue compuesta por 25 artículos, publicados desde 2017, con mayoría desarrollada en Japón (n=11); predominancia del método oscilométrico (n=22); los dispositivos más utilizados fueron de la marca Omron^®^ (n=14); el tipo de variabilidad más detectada fue la de largo plazo (n=10).

**Conclusiones:**

observado el creciente uso del método oscilométrico para análisis de la variabilidad presórica, presentando variedad de marcas y modelos de dispositivos automáticos.

## INTRODUCTION

The technological advance in blood pressure (BP) monitoring occurs due to the development of electronic devices for BP measurement and the progressive prohibition of the use of the mercury column in several countries^(1)^. The indirect measurement technique encompasses the auscultatory method, which may use mercury column or aneroid devices, and the oscillometric method, using electronic devices, which determine BP based on pressure oscillations of the cuff during inflation/disinflation induced by pulsatile blood flow in the compressed arteries^(2)^. Compared with other methods of BP measurement, oscillometric measurement has been the most widely used technology for BP measurement in developed countries due to advantages such as not being influenced by noise, it is a simple operation at low cost, and the possibility of taking several readings^(1)^.

The fact that it is possible to obtain multiple records by oscillometric measurement allows blood pressure variability (BPV) to be measured and monitored, defined as BP fluctuation during a given period under the influence of environmental factors, such as seasons, altitude, and stress; physical, such as posture or volemia; and emotional factors^(3-4)^. These fluctuations may occur in intervals of seconds or minutes, called very short-term BPV, generally assessed in the physician’s office. However, other variations may be found depending on the measurement interval: short-term BPV, assessed by 24-hour ambulatory BP monitoring (ABPM); medium-term BPV, assessed with home blood pressure monitoring (HBPM) between days; and long-term BPV, assessed between clinic visits over months or years, also called “visit-to-visit variation”^(4)^.

The BPV is recognized as a risk marker for organ damage, mortality, and cardiovascular events^(5)^. Its clinical significance is not fully established, but studies have shown an independent connection between different types of BPV and cardiovascular events and target-organ damages in individuals with arterial hypertension (AH) and those with diabetes *mellitus* (DM)^(3-4)^.

Diabetes is pointed out as a favorable scenario for BPV and identifying this variability can be a parameter for therapeutic adjustments, aiming to decrease its cardiovascular impact^(5)^. Studies indicate that the BPV in individuals with DM is a potential predictor of cardiovascular diseases (CVD) compared to people without diabetes^(6-7)^, explaining the more significant autonomic imbalance, increased arterial stiffness, and cardiovascular autonomic neuropathy^(7-8)^. Consequently, the assessment of pressure variability in clinical practice may optimize the prevention of CVD in this populace.

In this manner, the method and device of choice for BP measurement must be accurately defined^(9)^. However, due to the wide availability of devices in the market, researchers are concerned about the type of device used and its validation status^(10)^. Although there is current evidence that the method used for BP measurement and BPV assessment, as well as age and mid-level of BP, affect its magnitude^(11)^, no study has proposed to synthesize the methods and devices employed for BP measurement and assessment of each type of BPV in people with diabetes.

Given the gaps in the pertinent literature, it is considered relevant to produce this knowledge to encourage the BP measurement and BPV assessment in clinical practice through appropriate devices. Hence, we highlight the importance of this survey to outline primary studies on BPV in people with DM because it presents information on BP monitoring time, the number of measurements, techniques, and devices that have been used for each type of variability, as well as whether they are validated.

## OBJECTIVES

To map the methods and devices used to assess very short-term, short-term, medium-term, and long-term blood pressure variability in adults with type 1 or 2 DM.

## METHODS

### Ethical aspects

This research was not submitted to an ethics committee because it is a scoping review.

### Type of study

This study is a scoping review following the review method proposed by the Joanna Briggs Institute (JBI)^(12)^. Scoping reviews can be used to provide an overview of a subject and are valuable tools for recognizing evidence and identifying gaps in that evidence, as well as clarifying key concepts in a subject area^(12)^.

The research question was based on the strategy for a scoping review: Population, Concept, Context (PCC)^(12)^. It was defined: P - Adults and older people with DM type 1 or 2; C - Methods and devices used to measure pressure variability; C - In any setting (home, physician’s office, or an ambulatory). With that, the guiding question was established: which methods and devices are used to measure blood pressure variability (BPV) in people with diabetes *mellitus* type 1 or 2?

### Criteria of inclusion and exclusion

We included articles: available in full text and published in the last ten years (temporal frame adopted due to changes in the validation protocol of the devices reviewed by the European Society of Hypertension in 2010)^(13-14)^; without language restrictions and that analyzed arm blood pressure (BP); developed for adults aged 18 years or more^(15)^ and older adults with 60 years of age or more^(16)^; with type 1 or 2 DM; citing the type of method and device (brand and model) used for BP measurement. We also included articles that analyzed people with multiple diseases, including diabetes, provided that, when comparing the groups, the results were treated separately. Articles that did not respond to the study’s objective and used discontinued devices, i.e., no longer available for marketing, were excluded.

### Collection and organization of data

The literature search occurred in January and February 2021 and was conducted by a researcher in each database, with the keywords: Blood Pressure Variability and Diabetes in the National Library of Medicine (PubMed) and Web of Science databases. Searches were also performed in the Latin American and Caribbean Literature on Health Sciences (LILACS), Cumulative Index to Nursing and Allied Health Literature (CINAHL-EBSCO), Embase, and Scopus databases.

In order to adapt the search in the databases and platforms, were used the Health Science Descriptors (DeCs) in LILACS: Blood Pressure, Variability, Diabetes, and Diabetes *Mellitus*. In the English language databases, we used the Medical Subject Headings (MeSH) descriptors: Blood Pressure Variability, Blood Pressure, Variability, Diabetes *Mellitus*, and Diabetes. In CINAHL-EBSCO, were adopted the proper English terms: Diabetes *Mellitus*, Diabetes *Mellitus*, Type 2; Diabetes *Mellitus*, Type 1; Diabetes, Blood Pressure Variability (Chart 1). Besides the descriptors, the Boolean operators AND and OR were also used to help in the searches.

**Chart 1:** Literature search strategy, Fortaleza, Ceará, Brazil, 2021

LILACS*	PubMed**/ Web of science/Scopus/Embase	CINAHL** *
(Diabetes *Mellitus* OR Diabetes *Mellitus* Type 2 OR Diabetes *Mellitus* Type 1 OR Diabetes) AND (“Blood Pressure” OR “Blood Pressure” OR “Blood Pressure Variability”)	(Diabetes *Mellitus* OR Diabetes *Mellitus*, Type 2 OR Diabetes *Mellitus*, Type 1 OR Diabetes) AND (“Blood Pressure Variability”)	(Diabetes *Mellitus* OR Diabetes *Mellitus*, Type 2 OR Diabetes *Mellitus*, Type 1 OR Diabetes) AND (“Blood Pressure Variability”)

* *Latin American and Caribbean Literature in Health Sciences;*

** National Library of Medicine;

*** Cumulative Index to Nursing and Allied Health Literature.

After extraction from the databases, the articles were exported to the Zotero^®^ reference manager to remove duplicates. In the screening process, exploratory reading of titles and abstracts was performed by two independent researchers, with filters based on eligibility criteria, and those same researchers solved by consensus all the disagreements. The PRISMA extension for scoping reviews (PRISMA-ScR) was used to organize and present the summary of the articles’ selection^(17)^.

It stands out that the devices considered validated were those on the STRIDE BP sites of the European Society of Hypertension - International Society of Hypertension - World Hypertension League^(18)^, BIHS site of the British and Irish Hypertension Society^(19)^, JSH site of the Japanese Society of Hypertension^(20)^ and those found on the company’s site or a published study proving their validation.

Two independent reviewers extracted data from the included articles using a formulary developed by the researchers to map the title, author, journal, year of publication, country of origin, objective and delimitation, population, type of DM (1 or 2), place, type of variability (very-short, short, medium, and long-term), the method used for measuring BP; brand, model, and validation status of the device used for measuring BP. Subsequently, these data were entered into an Excel spreadsheet^®^.

### Data analysis

After treatment of the extracted data, the articles were characterized, and the results were grouped, synthesized, and described based on the research question through the elaboration of summary charts.

## RESULTS

**Figure 1:**
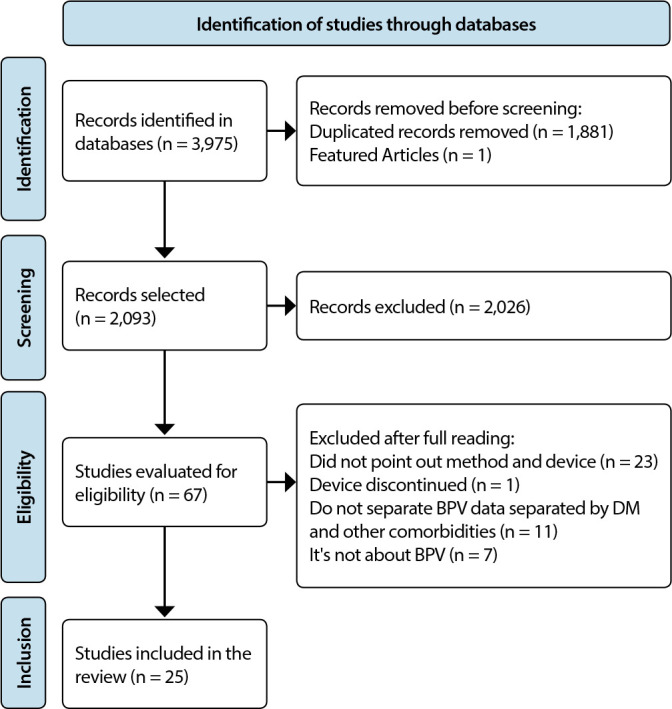
PRISMA Flowchart for scoping reviews (PRISMA-ScR) obtained from the literature search

A total of 3,795 articles were identified, of which 25 composed the final sample. The selection of articles was presented in the PRISMA Flowchart for scoping reviews (PRISMA-ScR), Figure 1.

Chart 2 presents the selected articles according to author/year/country, objective, delimitation, participants/type of DM, method, device, type of BPV/interval of measurements, and the number of measurements. As observed, most (60%) were published since 2016. As for the type of study, cross sectional was prevalent (44%). There was a predominance of type 2 DM, and Japan was the country that concentrated most (44%) of the publications. The study’s predominant type of BP variability was long-term BPV (40%).

**Chart 2:** Characteristics of publications, measurement methods, devices, and number of measurements (n = 25), Fortaleza, Ceará, Brazil, 2021

Main author/ /year/country	Objective	Study design/ participants /Type of DM	Method	Device	Type of BPV /interval of measurements
Najafi MT (2018)^(21)^ Iran	To investigate the association between microvascular and macrovascular diabetes complications and diurnal and nocturnal BP variability.	Cross-sectional/ 192 participants/ DM 2	Auscultatory Oscillometric	Mercury Sphygmomanometer Erkameter 3000^®^ ABPM Tiba Ambulo 2400^®^	Short-term/ Every 30 minutes for 24 hours ** One measurement at a time, every 30 consecutive minutes, for a period of 24 hours
Iuchi H (2017)^(22)^ Japan	To examine the association between daily BPV and two different types of ambulatorial BPV.	Cross-sectional/ 30 participants/ DM2	Oscillometric	TM-242\5; A&D (ABPM)^®^ *	Short- (24 hours) and medium- term (five days) One measurement every visit for five days One measurement every 60 minutes for 24 hours, during daytime (9:00 am to 9:00 pm) and night-time (00:00 am to 06:00 am)
Wan EYF (2017)^(23)^ China	To evaluate the connection between visit-to-visit variability of SBP and CVD/mortality risk in the Chinese DM2 population in primary care.	Retrospective cohort/ 124,105 participants/ type 2 DM	Oscillometric	TM-2655P, A&D Company^®^ UA-853, A&D Company^®^ M3A, EDAN^®^ *	Long-term (visit-to-visit) / two years Every three months (a total of nine)
Suzuki D (2020)^(24)^ Japan	To investigate the association between daily BPV at home and eTFG in individuals with diabetes and compare this connection with individuals without diabetes.	Cross-sectional J-HOP study/ 4,231 participants/ Type of DM not reported	Oscillometric	HEM-5001, Omron Healthcare^®^ *	Medium-term (visit-to-visit) / 14 days One measurement in the morning and at night
Veloudi P (2016)^(25)^ Australia	To determine the relation between BPV indices and retinal arteriolar diameter in non-diabetic participants and participants with type II diabetes.	Post-hoc analysis/ 35 non-diabetic and 28 DM2 participants	Oscillometric	TM-2430, A&D Medical^®^	Short-term/24 hours Every 20 minutes during the day and every 30 minutes at night
Papadopoulou E. (2020)^(26)^ Greece	To evaluate the effect of dapagliflozin on short-term BPV in patients with DM2.	Randomized clinical trial/ 43 participants in the dapagliflozin group and 42 in the placebo group/ type 2 DM	Oscillometric	Mobil-O-Graph, IEM-Stolberg^®^	Short-term/24 hours 20 minutes during the day (7am to 11pm) and every 30 minutes during the night (11pm to 7am).
Y. Gepner, Y (2016)^(27)^ Israel	To evaluate the effect of initiating moderate red wine consumption on 24-hour BP recordings and the effect of one common alcohol dehydrogenase (ADH) genetic variant among patients with type 2 diabetes.	Randomized clinical trial/ 224 patients/ DM2	Oscillometric	Oscar 2, SunTech Medical^®^	Short-term/24 hours (At the beginning and end of the study) Every 30 minutes during the day (6am to 11pm) and every 60 minutes at night (11pm to 6am)
Ushigome E (2014)^(28)^ Japan	Investigate the factors affecting BPV at home in patients with DM2.	Cross-sectional Multicenter study/ 1,114 participants: 608 males and 506 females/ type 2 DM	Oscillometric	HEM-70801C, Omron Healthcare^®^	Medium-term/ 14 days Three measurements in the morning and evening for 24 hours
Foo V (2017)^(29)^ Singapore	To determine whether HbA1c and SBP variability, assessed retrospectively based on regular, consecutive HbA1c and SBP values obtained over two years prior to the onset of moderate diabetic retinopathy, was independently associated with moderate retinopathy-specific diabetes.	Retrospective case-control study/ 398 participants/ DM2	Auscultatory	Aneroid Sphygmomanometer Series Six00; Accoson^®^ *	Long-term/two Years Three to five measurements every three months
Kalinga BE (2019)^(30)^ India	Compare the BPV with inflammation marker (Hs-CRP) to study the impact of the effect of BPV in patients with diabetes on vascular endothelium cells using 24-hour ABPM.	Case-control/ 100 Participants: 50 with DM2 and 50 without DM2	Oscillometric	Pressurometer P6; Del Mar Reynold^®^	Short-term/24 hours Every 30 min (7am to 10pm) and 60 min (10pm to 7am)
Ciobanu DM (2016)^(31)^ Romania	To evaluate the connection between high-sensitivity C-reactive protein (hsCRP) and BPV during 24-hour ambulatory BP monitoring in DM2 and healthy control individuals.	Cross-sectional/ 75 participants/ type 2 DM	Oscillometric	HolCard CR-07, Aspel^®^	Short-term/24h Every 30 min (7am to 10pm) and 60 min (10pm to 7am)
Fukui M (2013)^(32)^ Japan	To investigate the connection between BPV on one occasion and markers of arterial stiffness in patients with type 2 diabetes.	Cross-sectional Multicenter study/ 332 participants/ type 2 DM	Oscillometric	HEM-70801C, Omron Healthcare^®^	Medium-term/14 days Three measurements in the morning and night over 24 hours
Ciobanu DM (2019)^(33)^ Romania	To evaluate the connection between circulating adhesion molecules and ambulatory blood pressure variability in patients with type 2 diabetes and controls.	Cross-sectional/ 110 participants in two groups: controlled BP (n = 55) and non-controlled BP (n= 55)/Type 2 DM	Oscillometric	HolCard CR-07, Aspel^®^	Short-term/24h Every 30 min during the day (7am to 10pm) and every 60 min at night (10pm to 7am)
Matsumoto S (2014)^(34)^ Japan	To evaluate the reliability of home blood pressure (HBP) in patients with type 2 diabetes by comparing self-reported values with HBP measurements stored in the memory of the blood pressure (BP) monitor.	Cross-sectional/ 280 Participants/ type 2 DM	Oscillometric	HEM-7080IC, Omron Healthcare^®^	Medium-term/14 days Triplicate measurements in the morning and at night
Cardoso CRL (2020)^(35)^ Brazil	To investigate whether long-term visit-to-visit BPV (BP-VVV) impacts the prognosis for microvascular and macrovascular complications, developmental actions, and all-cause mortality.	Prospective cohort/ 632 participants/ type 2 DM	Oscillometric	HEM-907XL, Omron Healthcare^®^	Long-term (visit-to-visit) /24 months Three to four annual measurements
E. Ushigome (2018)^(36)^ Japan	To clarify whether daily home systolic blood pressure (HSBP) variability could have a significant prognostic role in the progression to macroalbuminuria in a prospective two-year study.	Prospective cohort/ 714 participants/ type 2 DM	Oscillometric	HEM-70801C, Omron Healthcare^®^	Medium-term/14 days. Triplicate measurements in the morning and at night.
T. Takao (2015)^(37)^ Japan	To determine whether visit-to-visit BPV can predict cardiovascular disease (CVD) incidence in patients with DM2, independent of mean BP, and to analyze the time-effect connection between BP and CVD risk..	Retrospective cohort/ 629 participants/ type 2 DM	Oscillometric	BP-10, Omron Healthcare^®^	Long-term (visit-to-visit) / 11 years **
Hashimoto, Y (2018)^(38)^ Japan	Investigate the connection between sarcopenia and blood pressure parameters, including BPV visit-to-visit to elderly patients with type 2 diabetes.	Cross-sectional with data from a cohort study/ 209 participants/ type 2 DM	Oscillometric	HEM-906, Omron Healthcare^®^ *	Long-term (visit-to-visit)/one year **
Ushigome E (2011)^(39)^ Japan	To investigate the connection between the variability of daily home blood pressure over 14 consecutive days and macroalbuminuria in patients with type 2 diabetes.	Cross-sectional Multicenter study/ 858 participants/ type 2 DM	Oscillometric	HEM-70801C, Omron Healthcare^®^	Medium-term (visit-to-visit) /14 days. Three measurements in the morning and at night for 14 days
Bhardwaj S (2014)^(40)^ Índia	To evaluate the 7-day/24-hour circadian pattern of BP and heart rate in diabetic patients to help diagnose and prevent cardiovascular morbidity.	Case-control/ 100 participants (50 males with type 2 DM and 50 males without diabetes)	Oscillometric	A&D TM-2430, A&D Company^®^	Short-term/24-hour BP for seven days Every 30 minutes during the day and 60-minute intervals during the night
Radaelli MG (2020)^(41)^ Italy	To retrospectively assess the coefficient of variation of mean SBP and its connection with CVD prevalence and risk of future CVD-related events using the ten-year UKPDS Risk Engine.	Cross-sectional, retrospective/ 970 medical charts	Oscillometric	Omron M6, Omron Healthcare^®^	Long-term/two years **
Okada H (2013)^(42)^ Japan	To investigate the connection between visit-to-visit variability in SBP and alteration in urinary albumin excretion (UAE) or development of albuminuria in patients with type 2 diabetes.	Retrospective cohort/ 354 patients/ Type 2 DM	Oscillometric	HEM-906, Omron Healthcare^®^ *	Long-term (visit-to-visit)/one year **
Noshad S (2014)^(43)^ Iran	To investigate whether variability in BP between visits is a significant predictor of progression to microalbuminuria independent of mean BP..	Retrospective cohort/ 194 medical charts/ DM2	Auscultatory	Riester Big Ben^®^, Jungingen *	Long-term/ 24 to 48 months **
Hsieh YT (2012)^(44)^ Taiwan	To evaluate the connection between all-cause mortality and blood pressure parameters (systolic blood pressure [SBP], diastolic blood pressure [DBP], pulse pressure [PP], mean arterial pressure [MAP]) and visit-to-visit variability in patients with type 2 diabetes.	Longitudinal cohort/ 2161 participants/ DM2	Oscillometric	HEM-1000, Omron Healthcare^®^ *	Long-term (visit-to-visit/two years **
Takao T (2014)^(45)^ Japan	To investigate whether visit-to-visit variability in systolic blood pressure (SBP) can predict the development and progression of diabetic nephropathy and retinopathy in patients with DM2.	Retrospective cohort/ 664 participants/ DM2	Oscillometric	BP-10, Omron Healthcare^®^	Long-term (visit-to-visit/sixteen years **

*BP - blood pressure; DM - diabetes mellitus; DM2 - type 2 diabetes mellitus; BPV - Variability of blood pressure; SBP - Systolic Blood Pressure; CVD - Cardiovascular Diseases; GFR - Glomerular Filtration Rate; HBA1c - Glycated Hemoglogin; DBP - Diastolic Blood Pressure;*

* Validation status not reported;

** Number of measurements not informed.

There was a prevalence of the oscillometric method for BP measurement (n = 22). Most of the devices were from the brand Omron^®^ (n = 14) and A&D^®^ (n = 5) and validated (n = 18), although, in some publications, the validation/approval status of some devices was not mentioned (n = 7).

It was possible to observe a variation in the number of readings depending on the measurement method. In short-term BPV assessment stood out the performance of one measurement every 30 minutes during the day and every 60 minutes at night (n = 4). As for the medium and long-term BPV, stood out the three measurements in the morning and at night (n = 5), as described in Chart 2.

## DISCUSSION

The literature mapping regarding the methods and devices for BP measurement and assessment of the types of BPV in DM patients allowed us to identify the predominance of long-term BPV (visit-to-visit), the oscillometric method, and the usage of several devices.

Automatic devices are gaining prominence and are progressively being used for BP measurement^(46)^. Thus, evidence suggests that automated recording seems to be the most promising approach because it provides relatively more accurate estimation, although it is still uncertain whether a specific device may be recommended as a standard product over another^(47)^. Therefore, there are many devices of various brands and models in the market.

As for the measurement method, the oscillometric has stood out for allowing a new approach to determine arterial stiffness, of which hypertension is an important cause and usually in association with diabetes. In those cases, the impact is even more extensive since it is a morbid condition due to its effects on the arteries^(48)^. Thus, individuals with diabetes have a more increased cardiovascular risk than the general population, so the importance of employing validated oscillometric devices for BP monitoring in this population has been emphasized.

The cardiovascular impacts of hypertension mainly depend on increased mean BP values and are independently associated with the increase of BPV, although its additional predictive value is unclear^(49)^. Furthermore, there are disagreements in the literature about which type of BPV is superior for estimating CVD risk^(50)^. There is also difficulty interpreting its impact because there is no gold standard device for BP measurement or specific guidelines for assessing its variability^(51)^. However, the studies highlighted the investigation of long-term BPV, performed visit-to-visit, in the occurrence of DM complications.

Although some disagreements, studies have emphasized the importance of assessing this type of variability, showing the association of long-term systolic blood pressure (SBP) variability with increased risk of all-cause mortality and complications in people with diabetes^(7)^. Although studies have highlighted the importance of visit-to-visit assessment of BPV in predicting cardiovascular disease, further research is still needed to determine the causes of its increase, its best estimate, and whether treatments improve clinical outcomes^(52)^. Moreover, its assessment may improve risk prediction beyond traditional risk factors and may be an important therapeutic target in patients with DM^(7)^.

According to the literature, each component of short-, medium-, or long-term BPV seems to be associated with important outcomes in the population in question. In this sense, studies indicate that the assessment of medium-term BPV by HBPM may assist in BP control and the prevention of nephropathy progression; and that the short-term, by ABPM, serves to evaluate the effects of autonomic neuropathy and the aspects of BP on sleep in patients with diabetes^(26)^. Consequently, the measurement methods and indices used to assess BPV should also be considered since the BP values depend on the time adopted as an interval and the choice of the estimation method^(52)^. It also stands out the necessity to ensure these measurements’ reliability in the physician’s office, the ambulatory, or at home. Each guideline establishes a minimum number of readings depending on the type of monitoring.

In this review, the number of measurements used to assess BPV did not follow a pattern, which could be explained by the lack of consensus on each type of BP monitoring recommendation. Regarding the recommendations of the protocols, in the case of the Brazilian guideline for BP monitoring, it is recommended that the device used for ABPM be programmed to measure BP at least every 30 minutes for 24 hours. For HBPM is recommended a measurement in the morning and at night, with three readings at each time, for a period of three to seven days^(53)^. The European guideline establishes a frequency of ABPM measurement every 20 or 30 minutes during the day and night, for 24 hours; and for HBPM, the frequency for measurements is twice in the morning and twice at night, for seven or at least three days^(50)^.

### Study limitations

Some limitations should be considered since the analysis was restricted to the studies in the databases as mentioned earlier; consequently, other studies possibly equally relevant to the research were not included. Another limiting factor was the absence of information in some studies, such as the time of BP monitoring, number, and interval of BP measurements. It should be highlighted that the scientific rigor of the studies was not evaluated.

### Contributions to the Fields of Nursing, Health or Public Policy

In synthesis, the scope review approach allowed us to identify the methods and devices, the validation status employed for BP measurement, and the types of BPV investigated in individuals with DM. Although the impact of this pressure variability is still little explored in people with diabetes, it is believed that its identification can be used as a parameter for therapeutic adjustments aiming to reduce cardiovascular damage in those individuals. Thus, these results may promote the BP measurement in clinical practice using appropriate devices and contribute to the delimitation of primary studies on BPV in people with DM.

## CONCLUSIONS

The current review mapped evidence that pointed to the increasing use of the oscillometric method with various brands and models of automatic devices, most of them validated. Moreover, the studies highlighted the long-term BPV, performed visit-to-visit. Furthermore, a variation in the number of measurements adopted was observed, which may be attributed to the lack of consensus on each type of BP monitoring recommendation. Thus, it is emphasized the need for further studies and standardization of the procedures for verification of BPV by international societies, recommending reliable measurement protocols.
